# Antioxidant Capacity of Rapeseed Extracts Obtained by Conventional and Ultrasound-Assisted Extraction

**DOI:** 10.1007/s11746-014-2557-4

**Published:** 2014-11-02

**Authors:** Aleksandra Szydłowska-Czerniak, Agnieszka Tułodziecka

**Affiliations:** Faculty of Chemistry, Nicolaus Copernicus University, 7 Gagarin Street, 87-100 Toruń, Poland

**Keywords:** Rapeseed, Extraction, Ultrasound, Antioxidant capacity

## Abstract

Ultrasound-assisted extraction (UAE) and conventional solid–liquid extraction were applied to extract total antioxidants from two rapeseed varieties. The antioxidant capacities (AC) of winter and spring rapeseed cultivars were determined by four different analytical methods: ferric reducing antioxidant power (FRAP), cupric reducing antioxidant capacity (CUPRAC), 2,2′-diphenyl-1-picrylhydrazyl (DPPH), 2,2′-azino-bis-3-ethylbenzothiazoline-6-sulfonic acid (ABTS). The average AC of the studied rapeseed cultivars ranged between 4.21–10.03 mmol Trolox (TE)/100 g, 7.82–10.61 mmol TE/100 g, 8.11–51.59 mmol TE/100 g, 22.48–43.13 mmol TE/100 g for FRAP, CUPRAC, DPPH and ABTS methods, respectively. There are positive correlations between total phenolics (TPC = 804–1625 mg sinapic acid (SA)/100 g) and AC of the studied rapeseed extracts (*r* = 0.2650–0.9931). Results of the principal component analysis (PCA) indicate that there are differences between the total amounts of antioxidants in rapeseed samples extracted by different extraction techniques. Rapeseed extracts obtained after 18 min of ultrasonication revealed the highest content of total antioxidants. The UAE is a very useful, efficient and rapid technique of oilseed samples preparation for determination of AC by different analytical methods.

## Introduction

Rapeseed (*Brassica napus*) is cultivated predominantly as winter or semi-winter form in Europe and Asia, respectively, whereas spring-sown canola types are more suited to the climatic conditions in Canada, northern Europe and Australia. It is known that, rapeseed gives a considerable yield of oil (around 45 %). Rapeseed cultivars classified as winter (requiring vernalization) and spring (without vernalization) differ in the vernalization requirements for flowering, which would affect the yielding and antioxidants content in seed and oil [1–4]. Rapeseed cultivars are a readily accessible source of natural antioxidants such as: polyphenols (sinapic acid derivatives are the predominant phenolics), tocopherols (α-tocopherol, γ-tocopherol and plastochromanol-8), sterols (mainly sitosterol, campesterol, brassicasterol, Δ5-avenasterol, stigmasterol), carotenoids and phospholipids [5–15]. Cultivars strongly reduced in erucic acid and glucosinolates (00 quality), but rich in bioactive compounds give one of the healthiest vegetable oils for human consumption. Antioxidants present in rapeseed and its products, have gained much attention due to their antioxidant capacities (AC) and free radical scavenging abilities, which potentially have beneficial implications in human health [6, 9, 12–14]. However, production of rapeseed oil with modified fatty acid composition and rich in antioxidants is possible through breeding of new double low rapeseed varieties and genetic engineering techniques. Improved knowledge on the AC analysis of rapeseed varieties would assist in modernization of technological process of rapeseed oil with high content of bioactive compounds.

Recently, different analytical methods including 2,2′-diphenyl-1-picrylhydrazyl (DPPH), ferric reducing antioxidant power (FRAP), reducing power, oxygen radical absorbance capacity (ORAC), cupric reducing antioxidant capacity (CUPRAC), β-carotene–linoleic acid, scavenging ability of hydrogen peroxide, electron spin resonance (ESR), enhanced chemiluminescence (ECL), photochemiluminescence (PCL) and silver nanoparticle-based assay (AgNP) were proposed for the AC evaluation of rapeseed cultivars [4–6, 9, 10, 12–15]. Moreover, individual phenolic compounds in rapeseed varieties were separated and quantified by chromatographic techniques [7, 11, 12], whereas total phenolics content (TPC) in rapeseed cultivars was determined by spectrophotometric method using Folin–Ciocalteu’s (FC) reagent [7, 8, 11–13, 16, 17]. Usually conventional methods such as: boiling, heating, conventional solid–liquid extraction (CSLE) have been applied for rapeseed samples preparation before analysis of individual and total phenolics and other antioxidants [4–7, 11–14, 16, 17]. All of them are rather labor intensive, time consuming, have low efficiency and high solvent consumption.

Recently ultrasound-assisted extraction (UAE) has been successfully used in food applications for extraction of bioactive compounds [18–23]. Among other new eco-friendly extraction techniques such as: enzyme-assisted extraction, microwave-assisted extraction, supercritical fluid extraction, pressurized liquid extraction, the UAE is the cheapest method and has the lowest instrumental requirements. Enhanced extraction efficiency of antioxidant compounds by ultrasound is attributed to the cavitation phenomenon produced in the solvent by the passage of an ultrasonic wave. Moreover, the UAE is recognized as an efficient extraction technique that reduces working times, increasing often the quality of the extracts [23]. Therefore, the UAE should be a matter of routine practice in analytical chemistry, which can use ultrasound energy for a different purposes in relation to sample preparation, mostly sample extraction. It is known, that over 80 % of analysis time is still spent on the sample preparation, and the UAE can speed up many procedures that are appropriate in other respects. The UAE was evaluated for isolation of antioxidant compounds from different matrices before AC determination [18–21]. However, to the best of our knowledge, there was no reference on the UAE application for the extraction of total antioxidants from rapeseed varieties before AC analysis. Only, Khattab et al. [8, 9] and Matthäus [10] extracted antioxidants from defatted oilseeds meal by ultrasonication for 1 and 45 min, respectively, but these authors did not investigate effect of ultrasonication time on the AC of rapeseed samples. Moreover, yield of total antioxidant compounds extracted from rapeseed by the CSLE and the UAE has not been compared.

In the present study, AC of extracts obtained from winter and spring rapeseed varieties by the CSLE and the UAE techniques were compared. Impact of extraction method and sonication time as well as rapeseed variety on the AC of the prepared extracts has been evaluated by means of four different analytical methods: FRAP, CUPRAC, DPPH, ABTS, whereas TPC in rapeseed samples was analyzed by FC assay. This report is the first comparative description of the UAE and the CSLE of antioxidant compounds from two rapeseed varieties.

Moreover, the FRAP, CUPRAC, DPPH, ABTS and TPC results were used as descriptors for principal component analysis (PCA) in order to differentiate the studied rapeseed samples and applied analytical methods.

## Materials and Methods

### Reagents

All reagents were of analytical or HPLC grade. 2,4,6-Tris(2-pyridyl)-s-triazine (TPTZ, 99 %), 2,9-dimethyl-1,10-phenanthroline (neocuproine), 2,2′-diphenyl-1-picrylhydrazyl radical (DPPH, 95 %), 2,2′-azino-bis(3-ethylbenzothiazoline-6-sulfonic acid) diammonium salt (ABTS), Folin–Ciocalteu’s reagent (FC reagent, 2 N), 6-hydroxy-2,5,7,8-tetramethylchromane-2-carboxylic acid (Trolox (TE), 97 %), sinapic acid (SA, 98 %), iron(III) chloride hexahydrate, sodium acetate, sodium carbonate, acetic acid, hydrochloric acid, ammonium acetate, copper(II) chloride, potassium persulfate, ethanol (96.0 %), methanol (99.8 %) were purchased from Sigma–Aldrich (Poznań, Poland). Redistilled water was used for the preparation of solutions.

### Plant Materials

Two black-seeded: winter (W) and spring (S) open pollinated rapeseed varieties of *Brassica napus* with a reduced content of glucosinolates (<10 μmol/g seed) and without erucic acid (double low, 00) were provided by a commercial supplier (Strzelce, Poland). Rapeseed samples in the original packing were stored in the dark at ambient temperature (22 ± 2 °C), until treatment and further analysis.

### Extraction of Antioxidants from Rapeseed Cultivars

All rapeseed samples were ground using a knife grinder (GRINDOMIX GM 200, Retsch, Haan, Germany) and sieved to a particle size of 0.5 mm.

### Conventional Solid–Liquid Extraction

A portion (2.0 g) of ground rapeseed and 20 mL of methanol–water (1:1 v/v) were transferred into round-bottom flasks and shaken using a shaker SHKA 2508-1CE (Labo Plus, Poland) at room temperature (22 ± 2 °C) for 30 min. Each sample was extracted in triplicate, and the residual rapeseed flour was separated by centrifugation (centrifuge MPW-310, Labo-Mix, Poland, 4,500 rpm, 15 min). The pooled extracts were filtered and stored in a refrigerator at 4 °C prior to analysis.

### Ultrasound-Assisted Extraction

A portion (2.0 g) of ground rapeseed and 15 mL of methanol–water (1:1 v/v) were transferred into an Erlenmeyer flask and placed in a ultrasonic clearer bath (5200DTD, Chemland, Poland) with a frequency of 40 kHz, ultrasound input power of 180 W and heating power of 800 W, equipped with a digital timer and temperature controller. The bottoms of the flasks were approximately 5 cm above the bottom of the bath. The solvent surface in the flasks was kept at the level of the water in the ultrasonic bath. Water in the ultrasonic bath was circulated and regulated at constant temperature (25 ± 0.3 °C) to avoid the water temperature increases as a result of exposure to ultrasound. The rapeseed samples were sonicated for 2, 6 and 10 min, respectively. The same rapeseed sample was extracted in triplicate and the residual rapeseed flour was separated by centrifugation (centrifuge MPW-310, Labo-Mix, Poland, 4,500 rpm, 15 min). The pooled extracts were filtered and stored in a refrigerator at 4 °C prior to analysis.

### Antioxidant Capacity Determination

#### FRAP Method

The AC of the studied rapeseed cultivars was determined by the spectrophotometric FRAP method according to procedure described previously [12]. In brief, freshly prepared FRAP reagent (2.5 mL of a 10 mmol/L TPTZ solution in 40 mmol/L HCl, 2.5 mL of 20 mmol/L FeCl_3_, and 25 mL of 0.1 mol/L acetate buffer, pH 3.6) was incubated at 40 °C for 15 min. Then, 0.03 mL of rapeseed extracts and 2 mL of FRAP reagent were transferred into a 10-mL volumetric flask and made up to volume with redistilled water. The blue solutions obtained were kept at 22 ± 2 °C for 20 min. The resulting absorbance was measured at 593 nm against a reagent blank (2 mL of FRAP reagent made up to 10 mL with redistilled water) using a Hitachi U-2900 spectrophotometer (Tokyo, Japan) in a 1-cm quartz cell.

Calibration curves were prepared using working solutions of TE in methanol between 1.00 × 10^−3^ and 1.80 × 10^−2 ^μmol/mL. The least-squares method was applied to calculate the line’s equation: *A*_593_ = (45.83 ± 0.44)*c*_TE_ + (0.012 ± 0.004) resulting in a determination coefficient, *R* ^2^ = 0.9998 and RSD_slope_ = 2.5 % (*n* = 5). The FRAP results were expressed in mmoL TE equivalents per 100 g of rapeseed. The within day precision of FRAP method was tested by analysis of sample containing 8.00 × 10^−3^ μmol TE/mL in five replicates. The obtained value of RSD = 4.4 % indicates reasonable repeatability of this assay. Moreover, the calculated detection (DL = 4.60 × 10^−4^ μmol TE/mL) and quantification (QL = 1.50 × 10^−3^ μmol TE/mL) limits confirm linearity concentration range for AC determination. The modified FRAP method appeared to be sensitive (*ε* = 5.00 × 10^4^ dm^3^ mol^−1^ cm^−1^).

#### CUPRAC Method

The spectrophotometric CUPRAC method was used for AC determination of rapeseed extracts according to Apak et al. [24] with minor modifications. In this procedure, 0.06–0.1 mL of extracts, 2 mL of 0.01 mol/L Cu(II), 2 mL of neocuproine solution (0.0075 mol/L) and 2 mL of ammonium acetate aqueous buffer (ammonium buffer was prepared by dissolving 19.27 g of ammonium acetate in 250 mL redistilled water) were transferred into a 10-mL volumetric flask and made up to volume with redistilled water. The obtained solutions were kept at 22 ± 2 °C for 30 min. The resulting absorbance was measured at 450 nm against a reagent blank (2 mL of 0.01 mol/L Cu(II), 2 mL of neocuproine solution and 2 mL of ammonium acetate aqueous buffer made up to 10 mL with redistilled water) using a Hitachi U-2900 spectrophotometer (Tokyo, Japan) in a 1-cm quartz cell.

Calibration curves were prepared using working solutions of TE in methanol between 6.00 × 10^−3^ and 6.00 × 10^−2^ μmol/mL. Five calibration curves were plotted using the least-squares method resulting in the equation: *A*_450_ = (14.85 ± 0.30)*c*_TE_ −(0.022 ± 0.011), *R* ^2^ = 0.9990 and RSD_slope_ = 1.44 %. However, RSD = 1.8 % (*n* = 5) for TE concentration = 3.00 × 10^−2^ μmol TE/mL illustrates reasonable repeatability of this method, whereas the calculated DL = 3.00 × 10^−3^ μmol TE/mL and QL = 1.00 × 10^−2^ μmol TE/mL as well as *ε* = 1.40 × 10^4^ dm^3^ mol^−1^ cm^−1^ confirm linearity of concentration range and sensitivity of the proposed CUPRAC assay. The CUPRAC results were expressed in mmoL TE equivalents per 100 g of rapeseed.

#### DPPH Method

The modified DPPH method was used for AC determination of rapeseed varieties [12, 13]. In brief, 0.01–0.02 mL of 50 % methanolic rapeseed extracts was added to 1.99–1.98 mL of methanol and 0.5 mL of DPPH methanolic solution (304.0 μmol/L). The mixture was shaken vigorously and left in darkness for 15 min. The absorbance was measured at 517 nm against a reagent blank (2 mL of methanol + 0.5 mL of DPPH methanolic solution) using a Hitachi U-2900 spectrophotometer (Tokyo, Japan) in a 1-cm quartz cell.

The scavenging of DPPH was calculated as follows:  %DPPH scavenging = [(A_control_ − A_sample_)/A_control_] × 100, where A_control_ = absorbance of DPPH radical + methanol; A_sample_ = absorbance of DPPH radical + standard (or seed extract).

Calibration curves were prepared using working solutions of TE between 2.00 × 10^−2^ and 1.00 × 10^−1^ μmol/mL. Five calibration curves were plotted using the least-squares method resulting in equation:  %DPPH = (668.9 ± 12.2)*c*_TE_ + (2.36 ± 0.80), *R* ^2^ = 0.9988 and RSD_slope_ = 4.8 %. However, DPPH values expressed as milimoles of TE equivalents per 100 g of rapeseed samples were obtained from the following linear relationship:  %DPPH = *f*(*c*_TE_). The repeatability of DPPH method (RSD = 2.4 %) was tested by analyzing (*n* = 5) solution of TE (6.00 × 10^−2^ μmol TE/mL). The calculated DL = 4.30 × 10^−3^ μmol TE/mL and QL = 1.40 × 10^−2^ μmol TE/mL for standard methanolic solutions of TE, confirm linearity concentration range for AC determination of the investigated samples. The DPPH assay (*ε* = 2.40 × 10^3^ dm^3^ mol^−1^ cm^−1^) appeared to be the least sensitive method used for determination of AC.

#### ABTS Method

The spectrophotometric ABTS method was used for AC determination of rapeseed extracts according to Chavan et al. [18] with minor modifications. In the first step, ABTS radical cation (ABTS^•+^) was produced by reacting 7 mmol/L ABTS stock solution with 2.45 mmol/L potassium persulfate at a ratio of 1:0.5 and the mixture was kept in darkness at 22 ± 2 °C for 12–16 h before use. For the study of rapeseed extracts, the ABTS^•+^ solution was diluted with ethanol to an absorbance of 0.70 (±0.02) at 734 nm. Briefly, 0.01 mL of methanolic extract was added to 2.49 mL of ABTS^•+^ solution and the mixture was incubated at 30 °C for 1 min. The absorbance was measured at 734 nm against a reagent blank (2.5 mL of ABTS^•+^ solution) using a Hitachi U-2900 spectrophotometer (Tokyo, Japan) in a 1-cm quartz cell.

The scavenging of ABTS was calculated as follows:  %ABTS scavenging = [(A_control_ − A_sample_)/A_control_] × 100, where A_control_ = absorbance of ABTS^•+^; A_sample_ = absorbance of ABTS^•+^ + standard (or seed extract).

Calibration curves were prepared using working solutions of TE in methanol between 1.00 × 10^−2^ and 1.50 × 10^−1^ μmol/mL. Five calibration curves were plotted on the same day. The least-squares method was applied to calculate the line’s equation:  %ABTS = (369.1 ± 12.0)*c*_TE_ + (10.3 ± 1.1) (RSD_slope_ = 0.8 %) resulting in a determination coefficient *R* ^2^ = 0.9974. The ABTS values expressed as milimoles of TE equivalents per 100 g of rapeseed samples were obtained from the following linear relationship:  %ABTS = *f*(*c*_TE_). The within day precision of ABTS method was evaluated by analysis of sample containing 5.00 × 10^−2^ μmol TE/mL in five replicates. The value of RSD = 3.5 % indicates reasonable repeatability of the used method. However, the calculated DL = 1.30 × 10^−2^ μmol TE/mL and QL = 4.20 × 10^−2^ μmol TE/mL confirm the linearity concentration range for ABTS determination of the investigated samples. The proposed ABTS method appeared to be sensitive (*ε* = 4.80 × 10^3^ dm^3^ mol^−1^ cm^−1^).

### Total Phenolics Content Determination

TPC was determined spectrophotometrically using the FC reagent, according to a procedure described previously [14]. Briefly, 0.1 mL of methanolic extract and 0.5 mL of FC reagent were transferred into a 10 mL calibration flask. The mixture was hand shaken for 3 min, and 1 mL of saturated sodium carbonate solution (22.0 %) was added and made up to the mark with redistilled water. After 1 h, solutions were centrifuged at 10,000 rpm for 15 min (centrifuge MPW-310, Labo-Mix, Poland), and the absorbance at 725 nm was measured against a reagent blank (0.5 mL of FC reagent + 1 mL of saturated sodium carbonate solution made up to 10 mL with redistilled water) using a Hitachi U-2900 spectrophotometer (Tokyo, Japan).

The calibration plot, *A*_725_ = (7.14 ± 0.18)*c*_SA_ + (0.022 ± 0.012) was linear (*R* ^2^ = 0.9966) in the concentration range between 0.88 and 13.2 μg/mL for methanolic solutions of SA. The values of RSD_slope_ = 1.3 % (*n* = 5), RSD = 1.6 % for *c*_SA_ = 6.6 μg/mL (*n* = 5) and *ε* = 2.00 × 10^4^ dm^3^ mol^−1^ cm^−1^ indicate reasonable within-day precision and sensitivity of this method. Moreover, the obtained results of DL = 0.87 μg SA/mL and QL = 2.90 μg SA/mL confirm linearity concentration range for determination of TPC by FC method. TPC in rapeseed varieties was expressed as mg SA equivalents per 100 g of rapeseed samples.

### Statistical Analysis

The AC and TPC in the studied rapeseed cultivars were determined (five portions of each extract obtained by the CSLE and the UAE techniques were analyzed within 1 day) by the FRAP, CUPRAC, DPPH, ABTS and FC methods, respectively. The obtained results were presented as: means *c* ± standard deviations (SD). Moreover, the Pearson correlation test was used to calculate the correlations between AC determined by different analytical methods and TPC in rapeseed extracts. Differences of *p* < 0.05 were considered significant. One-way analysis of variance (ANOVA), followed by the Duncan test, was performed to analyze the significant differences between data (*p* < 0.05). PCA was employed to study clustering and differentiation of the obtained extracts of two rapeseed varieties on the basis of FRAP, CUPRAC, DPPH, ABTS and TPC results. The scores and loadings of the data analyzed by PCA were displayed as bi-plot. The chemometric analyses were performed using the Statistica (Windows software package, version 8.0).

## Results and Discussion

### Determination of Antioxidant Capacity of Rapeseed Extracts

The experimental values of FRAP, CUPRAC, DPPH, ABTS and TPC in winter and spring rapeseed cultivars are listed in Table 1.

**Table 1:** Antioxidant capacities of rapeseed extracts obtained by the CSLE and the UAE methods

Analytical method/ rapeseed cultivar	Conventional extraction	Ultrasound-assisted extraction		
	*t* = 90 min	*t* = 6 min	*t* = 18 min	*t* = 30 min
Antioxidant capacity				
FRAP^A^ ± SD (mmol TE/100 g)				
Winter	5.13 ± 0.22^b,x^	6.89 ± 0.16^a,y^	10.03 ± 0.26^b,z^	10.01 ± 0.13^b,z^
Spring	4.21 ± 0.19^a,x^	6.29 ± 0.20^a,y^	8.13 ± 0.30^a,z^	8.10 ± 0.30^a,z^
CUPRAC^A^ ± SD (mmol TE/100 g)				
Winter	8.87 ± 0.21^d,x^	9.29 ± 0.14^c,y^	10.61 ± 0.22^b,z^	10.50 ± 0.38^b,z^
Spring	7.82 ± 0.072^c,x^	8.13 ± 0.25^b,y^	8.98 ± 0.17^a,b,z^	8.91 ± 0.13^a,z^
DPPH^A^ ± SD (mmol TE/100 g)				
Winter	10.67 ± 0.40^e,x^	23.04 ± 0.91^e,y^	51.59 ± 0.92^e,z^	51.49 ± 0.44^e,z^
Spring	8.11 ± 0.28^c,x^	20.51 ± 1.01^d,y^	34.21 ± 1.47^c,z^	34.13 ± 0.91^c,z^
ABTS^A^ ± SD (mmol TE/100 g)				
Winter	22.48 ± 1.02^f,x^	25.51 ± 1.11^f,y^	34.46 ± 1.50^c,z^	34.40 ± 1.03^c,z^
Spring	26.73 ± 0.93^g,x^	28.65 ± 1.41 ^g,x^	43.13 ± 1.08^d,y^	43.11 ± 1.03^d,y^
Total phenolic content				
TPC^A^ ± SD (mg SA/100 g)				
Winter	804 ± 39^h,x^	1237 ± 33^h,y^	1530 ± 52^h,z^	1518 ± 68 ^h,z^
Spring	1201 ± 44^i,x^	1353 ± 60^i,y^	1625 ± 38^i,z^	1616 ± 50^i,z^

Different letters within the same column indicate significant differences between AC determined by four analytical methods (a–g) and TPC (h–i) in winter and spring rapeseed varieties. Different letters (x–z) within the same row indicate significant differences between FRAP, CUPRAC, DPPH, ABTS, respectively and TPC in extracts of each rapeseed variety prepared by the CSLE and the UAE for different time (one-way ANOVA and Duncan test, *p* < 0.05)

^A^Values are means ± standard deviations, *n* = 5

It can be noted that the AC of rapeseed extracts determined by the modified analytical procedures differ significantly from each other (Duncan test). Also, significant differences for mean TPC values were observed between winter and spring rapeseed varieties (Table 1). This variability can be explained by the influences of: (1) conventional extraction and UAE at different extraction time, (2) analytical parameters of the applied methods and (3) genetic, agronomic and environmental factors, which would affect the level of antioxidants.

It is evident that the winter rapeseed variety revealed higher FRAP, CUPRAC and DPPH values than the spring rapeseed variety, whereas extracts of the spring rapeseed cultivar had higher AC determined by the ABTS method, probably as a result of a higher TPC with high molecular weight (Table 1). This fact indicated that the ABTS^•+^ free radical is more sensitive to high molecular weight phenolic compounds such as condensed tannins, which are the major bioactive contributors to ABTS values of the studied rapeseed extracts. Results of AC and TPC in rapeseed samples obtained by the UAE during 18 min were significantly higher than by the CSLE for 90 min (Table 1). The UAE permits higher extraction yields in shorter period of time, thereby reducing the electrical energy input. The studied extracts prepared by ultrasonic treatment for 18 min had about two times higher results of FRAP (10.03 and 8.13 mmol TE/100 g) and ABTS (34.46 and 43.13 mmol TE/100 g) than samples obtained by the CSLE for 90 min (FRAP = 5.13 and 4.21 mmol TE/100 g, ABTS = 22.48 and 26.73 mmol TE/100 g for winter and spring varieties, respectively). However, DPPH values (51.59 and 34.21 mmol TE/100 g) of rapeseed samples (time of the UAE = 18 min) were above 4 times higher in comparison with DPPH results for the extracts of winter (10.67 mmol TE/100 g) and spring (8.11 mmol TE/100 g) cultivars not treated by ultrasounds. Similar, 18 min of sonication resulted in the highest TPC in extracts of two rapeseed varieties (Table 1). The results obtained indicate that the ultrasonication time of 18 min yielded the highest AC and TPC from rapeseed cultivars. Values of FRAP, CUPRAC, DPPH, ABTS and TPC in rapeseed extracts decreased insignificantly at the UAE time of 30 min. Prolonging time of ultrasound treatment was not able to increase the AC and TPC in rapeseed extracts. Therefore, the UAE time longer than 18 min an insignificantly decreased the extraction yield of antioxidant compounds. Hence, 18 min is suitable time duration for the extraction of total antioxidants from winter and spring rapeseed cultivars. Similar impact of sonication time on TPC and other antioxidants yield from various plants and fruits were observed by Chavan and Singhal [18], Khan et al. [19], Le and Le [20] and Porto et al. [21].

On the other hand, CUPRAC values (7.82–10.61 mmol TE/100 g) were higher than FRAP values (4.21–10.03 mmol TE/100 g) for extracts of two rapeseed varieties (Table 1). There were no significant differences in FRAP and CUPRAC values of samples prepared by the UAE for 18 and 30 min. This fact can be explained, that CUPRAC and FRAP assays are based on the same reaction mechanism and measure the ability of rapeseed antioxidants to transfer one electron to reduce copper and iron ions, respectively, which form the colored complexes with ligands. However, DPPH (8.11–51.59 mmol TE/100 g) and ABTS values (22.48–43.13 mmol TE/100 g) for all samples were about 2 and 5 times higher in comparison with CUPRAC and FRAP results (4.21–10.61 mmol TE/100 g). Also, DPPH and ABTS assays are classified as a single electron transfer reactions, although these methods are based on the measurement of the reducing ability of rapeseed antioxidants toward colored radical DPPH^•^ and radical cation ABTS^•+^. However, only an insignificant differences between DPPH results for samples of spring rapeseed variety and ABTS values for extracts from winter rapeseed variety sonicated during 18 and 30 min, respectively were observed. Moreover, Duncan test indicated that extracts prepared from winter and spring rapeseed varieties with ultrasound treatment at *t* = 6 min did not differ significantly in FRAP results. Also an insignificant differences in CUPRAC (7.82 mmol TE/100 g) and DPPH (8.11 mmol TE/100 g) results for spring rapeseed extracts obtained by the CSLE were found.

On the other hand, there were significant differences (Duncan test *p* < 0.05) in FRAP, CUPRAC, DPPH, ABTS (except spring variety ultrasound treated for 6 min), respectively and TPC for 50 % methanolic extracts of each rapeseed cultivar obtained after the CSLE and the UAE (Table 1). It can be noted that ultrasonication significantly increased the results of FRAP (34–96 %), CUPRAC (4–20 %), DPPH (116–384 %), ABTS (7–61 %) and phenolics extraction yield (13–90 %) from two rapeseed cultivars compared to the CSLE. Although ABTS values differed insignificantly between extracts of spring rapeseed variety obtained after the CSLE and 6 min of sonication. However, ultrasonic application allows extraction of total antioxidants in a time (6 min) much shorter than that required by the classical method (90 min). The ultrasound treatment of rapeseed samples can increase extraction of total antioxidants, while significantly reducing extraction time, thus improving overall efficiency.

It is noteworthy that FRAP (6.29–10.03 mmol TE/100 g) and DPPH (20.51–51.59 mmol TE/100 g) values of winter and spring rapeseed cultivars treated by ultrasound were higher than FRAP (3.31–7.64 mmol TE/100 g) and DPPH (3.32–7.65 mmol TE/100 g) results for rapeseed samples obtained by the CSLE in our previous reports [12, 13]. Although, the discussed rapeseed extracts revealed about 43 times lower CUPRAC values (7.82–8.98 mmol TE/100 g and 8.87–10.61 mmol TE/100 g for spring and winter cultivars, respectively) than methanolic extracts of spring (CUPRAC = 352 mmol TE/100 g) and winter (CUPRAC = 418 mmol TE/100 g) rapeseed cultivars obtained by the CSLE [4]. Moreover, different amounts (0.01–1 mg) of rapeseed extracts prepared by the UAE technique were used to scavenge the DPPH radicals by 50 % [9, 10]. However, the results obtained of TPC (804–1625 mg SA/100 g) in winter and spring rapeseed varieties are similar to those (756–1821 mg SA/100 g) reported by other authors [8, 12, 13, 16], whereas higher amounts of TPC in different rapeseed cultivars (1506–3524 mg SA/100 g) were determined by Cai and Arntfield [7], Siger et al. [11] and Liu et al. [17].

The values of RSD ranged between 1.3–4.5, 0.9–3.6, 0.9–4.9, 2.4–4.9 and 2.3–4.9 %, respectively, indicating reasonable repeatability of the modified FRAP, CUPRAC, DPPH, ABTS and FC assays. Similar RSD values of FRAP (1.0–4.4 %), CUPRAC (2.3–2.6 %), DPPH (0.2–3.4 %), and FC (0.5–5.3 %) for different rapeseed varieties were reported by others [4, 11–14, 16].

### Correlation Between Antioxidant Capacities and Total Phenolics Content in Rapeseed Varieties

Regression analysis was performed for the correlations among AC and TPC in rapeseed extracts prepared by the CSLE and the UAE techniques (Table 2).

**Table 2:** Correlation coefficients (*r*) between TPC and AC of rapeseed extracts determined by four different analytical methods

	FRAP	CUPRAC	DPPH	ABTS
TPC	0.7443^*^	0.3680	0.7524^*^	0.8893^*^
ABTS	0.6370	0.2650	0.6289	
DPPH	0.9931^**^	0.8766^*^		
CUPRAC	0.8791^*^			

* *p* < 0.05, ** *p*  < 0.0001

It can be noted that the results of TPC in the studied rapeseed extracts analyzed by FC assay correlated significantly positively with their AC determined by FRAP (*r* = 0.7443), DPPH (*r* = 0.7524) and ABTS (*r* = 0.8893) methods. Also, significant linear correlations (*r* = 0.9149–0.9468 and *r* = 0.6833–0.9516) between TPC–FRAP and TPC–DPPH values of various rapeseed varieties were demonstrated in our previous papers [12–14]. However, higher correlation coefficient for the relationship between TPC and the reducing power of spring variety canola type Cyclone hull (*r* = 0.966) was reported by Amarowicz et al. [5]. In these cases, high phenolics content is an important factor in determining the AC of rapeseed. On the other hand, there was no significant correlation between TPC and their ability to copper(II) ions reduction (Table 2). It has been suggested that the AC of rapeseed extracts determined by CUPRAC assay is more related to the kind of phenolic compounds present in extracts than their total content. Therefore, not all of the phenolic compounds are active reducers of copper(II) ions or have the same matrix effect. Also, Yoshie-Stark et al. [15] and Matthäus [10] did not find a linear correlations between TPC and AC of rapeseed cultivars determined by DPPH, β-carotene bleaching and ESR methods (*r* = 0.0117, 0.0092 and 0.0079). Therefore, the total phenolics assay may not be a suitable candidate for measuring the AC of every sample.

It is noteworthy that there are significant (*p* < 0.05 and *p* < 0.0001), positive correlations between FRAP–DPPH, CUPRAC–DPPH and FRAP–CUPRAC methods used to determine the AC of the studied rapeseed samples (correlation coefficients ranged between 0.8766 and 0.9931). Similar correlation coefficients (*r* = 0.8199–0.9999) for the relationship between FRAP and DPPH results of different rapeseed varieties were found previously [12–14]. However, the AC determined by the ABTS assay and those results obtained from the FRAP, CUPRAC and DPPH methods were not correlated significantly (*r* = 0.2650–0.6370), which implied that antioxidants in these extracts were not capable of scavenging radical cations (ABTS^•+^) and reducing oxidants (cupric and ferric ions) or DPPH^•^ radical. Moreover, ABTS^•+^ is an N-centered radical with sterically limited access to polymeric antioxidant compounds giving rise to slow reactions. For comparison, similar correlation coefficients for the relationship between ABTS–CUPRAC (*r* = 0.5886 and 0.4497), ABTS–DPPH (*r* = 0.4464 and 0.7491), as well as ABTS–FRAP (*r* = 0.2963 and 0.2907) for dialyzed and non-dialyzed fraction after intestinal digestion of processed tomatoes were calculated by Kamiloglu et al. [25].

### Principal Component Analysis

**Fig. 1:**
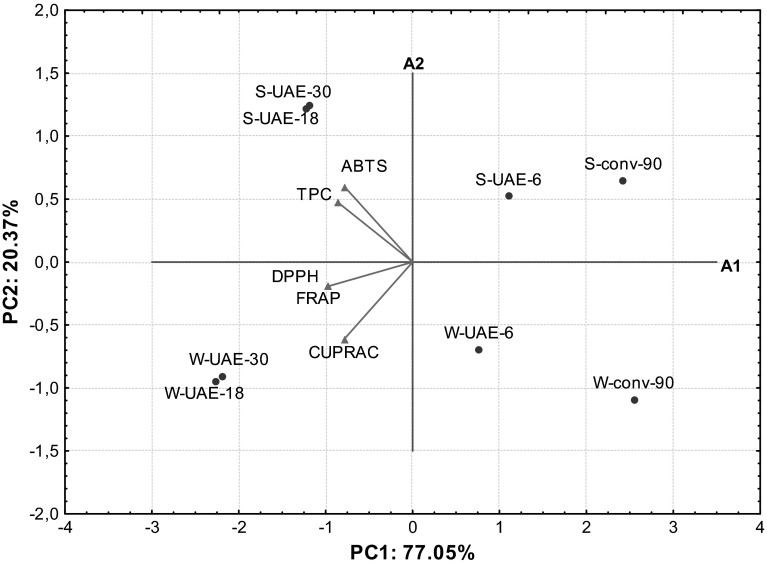
Biplot of scores and loadings of data obtained from FRAP, CUPRAC, DPPH, ABTS and TPC results for extracts of two rapeseed cultivars prepared by the CSLE and the innovative UAE

PCA was applied to observe any possible groups within extracts of two rapeseed varieties obtained by the CSLE and the innovative UAE technique. A set of five orthogonal variables (PC) was generated by PCA. The first (PC1) and second (PC2) principal components had a high eigenvalues (3.85 and 1.02, respectively) and accounted for 77.05 and 20.37 % of the variability in the data set. The remaining three generated PC (PC3, PC4 and PC5) yielded progressively lower eigenvalues (<1; 0.11, 0.014 and 0.0060, respectively) and did not explain the variability in the data. Therefore, only the first two PC were used for further study. The PC1 inversely correlated with all variables: FRAP (−0.9774), CUPRAC (−0.7836), DPPH (−0.9771), ABTS (−0.7730) and TPC (−0.8549), whereas PC2 was highly contributed by CUPRAC (−0.6111) and ABTS (0.5898). Evidently, PC1 is generally more correlated with the variables than PC2. The distribution of the most significant variables (FRAP, CUPRAC, DPPH, ABTS and TPC results) along the two first PC and the groupings and/or the differences among extracts of winter and spring rapeseed varieties prepared by different extraction techniques were presented in the bi-plot (Fig. 1).

It is noteworthy that ABTS and TPC were the variables with negative loadings on PC1 and positive loadings on PC2. However, CUPRAC, FRAP and DPPH were features with negative loading on PC1 and PC2. The PCA graph revealed that the 50 % methanolic extracts of spring and winter rapeseed cultivars obtained after 18 and 30 min of the UAE with high antioxidants content were located to the left in the score bi-plot and had negative values for PC1. However, extracts prepared in a shorter time of the UAE (6 min) and by the mechanical stirring of rapeseed samples (90 min) with low AC were situated at the right in the diagram and had positive values for PC1 (Fig. 1). Furthermore, extracts of winter rapeseed variety with higher FRAP, CUPRAC and DPPH values than spring rapeseed samples were located under the A1 axis. However, 50 % methanolic extracts of spring rapeseed cultivar with higher ABTS and TPC results were situated above the A1 axis. Impact of different techniques and sonication time on efficiency of antioxidants extraction from the studied rapeseed cultivars was observed on PCA graph (Fig. 1). The extracts of winter and spring rapeseed cultivars prepared during the CSLE for 90 min with the lowest values of FRAP, CUPRAC, DPPH, ABTS and TPC were separated from other studied samples. However, ultrasound treated rapeseed samples for 6 min with significantly higher total antioxidants content (except ABTS value for spring rapeseed cultivar) evidently created a distinct cluster. Moreover, there is very short distance between rapeseed samples prepared by the UAE for 18 and 30 min. This can be explained by the fact that AC and TPC in these extracts did not differ significantly (Duncan test, Table 1). The formed groups of extracts generally have similar AC determined by the modified FRAP, CUPRAC, DPPH, ABTS methods and TPC. It is noteworthy that there are differences in the total amount of antioxidants extracted by different techniques under various conditions from the same rapeseed samples.

## Conclusions

The UAE of rapeseed varieties produced higher recoveries of total antioxidants in comparison with the CSLE. An ultrasonication time of 18 min yielded the highest AC and TPC from winter and spring rapeseed cultivars. Application of ultrasound allowed extraction of total antioxidants in a time much shorter (18 min) than by the CSLE (90 min), whereas the yield of antioxidant extraction was increased by about 1.5–5 times. Moreover, the results of PCA indicated that conditions of different extraction techniques had a significant influence on the total antioxidants amounts of two rapeseed cultivars. The proposed UAE procedure of rapeseed sample preparation required a shorter treatment time and smaller amounts of reagents than the CSLE, thus significantly reducing potential environmental contamination. The UAE appears to have great potential as a technique for the extraction of antioxidants from rapeseed varieties, whereas the proposed analytical methods are relatively simple, precise and convenient for the determination of AC and TPC in rapeseed extracts. Moreover, there are positive correlations between TPC and AC of the studied rapeseed extracts determined by different analytical methods. On the other hand, the higher level of rapeseed antioxidants obtained during the UAE in comparison with those extracted by the CSLE, is of major interest from fat industrial point of view, because solvent amounts were reduced and extraction times shortened.

## Abbreviations

A: Absorbance
AC: Antioxidant capacity/capacities
ABTS: 2,2′Azino-bis-3-ethylbenzothiazoline-6-sulfonic acid
AgNP: Silver nanoparticle-based assay
ANOVA: Analysis of variance
c: Concentration
CSLE: Conventional solid–liquid extraction
CUPRAC: Cupric reducing antioxidant capacity
DL: Detection limit
DPPH: 2,2′-Diphenyl-1-picrylhydrazyl
ε: Molar extinction coefficient
ECL: Enhanced chemiluminescence
ESR: Electron spin resonance
FC: Folin–Ciocalteu
FRAP: Ferric reducing antioxidant power
ORAC: Oxygen radical absorbance capacity
QL: Quantification limit
PC: Principal component
PCA: Principal component analysis
PCL: Photochemiluminescence
r: Correlation coefficient
R^2^: Determination coefficient
RSD: Relative standard deviation
SA: Sinapic acid
SD: Standard deviation
TE: Trolox(6-Hydroxy-2,5,7,8-tetramethylchromane-2-carboxylic acid)
TPC: Total phenolics content
TPTZ: 2,4,6-Tris(2-pyridyl)-s-triazine
UAE: Ultrasound-assisted extraction
